# Clinician and Health Care Leaders' Experiences with—and Perceptions of—COVID-19 Documentation Reduction Policies and Practices

**DOI:** 10.1055/s-0041-1739518

**Published:** 2021-11-24

**Authors:** Amanda J. Moy, Jessica M. Schwartz, Jennifer Withall, Eugene Lucas, Kenrick D. Cato, S. Trent Rosenbloom, Kevin Johnson, Judy Murphy, Don E. Detmer, Sarah Collins Rossetti

**Affiliations:** 1 Department of Biomedical Informatics, Columbia University, New York, New York, United States; 2 Columbia University School of Nursing, New York, New York, United States; 3 NewYork-Presbyterian Hospital, New York, New York, United States; 4 Department of Emergency Medicine, Columbia University Irving Medical Center, New York, New York, United States; 5 Department of Biomedical Informatics, Vanderbilt University, Nashville, Tennessee, United States; 6 Twin Cities, Minnesota, United States; 7 Department of Public Health Sciences, University of Virginia, Charlottesville, Virginia, United States

**Keywords:** electronic health records, documentation burden, physician, nurse, survey

## Abstract

**Background** Substantial strategies to reduce clinical documentation were implemented by health care systems throughout the coronavirus disease-2019 (COVID-19) pandemic at national and local levels. This natural experiment provides an opportunity to study the impact of documentation reduction strategies on documentation burden among clinicians and other health professionals in the United States.

**Objectives** The aim of this study was to assess clinicians' and other health care leaders' experiences with and perceptions of COVID-19 documentation reduction strategies and identify which implemented strategies should be prioritized and remain permanent post-pandemic.

**Methods** We conducted a national survey of clinicians and health care leaders to understand COVID-19 documentation reduction strategies implemented during the pandemic using snowball sampling through professional networks, listservs, and social media. We developed and validated a 19-item survey leveraging existing post-COVID-19 policy and practice recommendations proposed by Sinsky and Linzer. Participants rated reduction strategies for impact on documentation burden on a scale of 0 to 100. Free-text responses were thematically analyzed.

**Results** Of the 351 surveys initiated, 193 (55%) were complete. Most participants were informaticians and/or clinicians and worked for a health system or in academia. A majority experienced telehealth expansion (81.9%) during the pandemic, which participants also rated as highly impactful (60.1–61.5) and preferred that it remain (90.5%). Implemented at lower proportions, *documenting only pertinent positives to reduce note bloat* (66.1 ± 28.3), c *hanging compliance rules and performance metrics to eliminate those without evidence of net benefit* (65.7 ± 26.3), and *electronic health record (EHR) optimization sprints* (64.3 ± 26.9) received the highest impact scores compared with other strategies presented; support for these strategies widely ranged (49.7–63.7%).

**Conclusion** The results of this survey suggest there are many perceived sources of and solutions for documentation burden. Within strategies, we found considerable support for telehealth, documenting pertinent positives, and changing compliance rules. We also found substantial variation in the experience of documentation burden among participants.

## Background and Significance

The widespread adoption and use of electronic health records (EHRs) coupled with a simultaneous increase in regulatory demands have led to a national epidemic of documentation burden among clinicians, including physicians, nurses, and other health professionals. 1 Physicians in the ambulatory setting spend nearly half their workday in the EHR—the majority of which on administrative and clerical tasks involving clinical documentation. 2 3 Nurses spend approximately one-quarter 4 to over one-third 5 6 of their EHR time on documentation. In the acute care setting, nurses document approximately one data point per minute. 7 There is now scientific consensus that high documentation times and documentation-related stress are associated with clinician burnout, increased medical errors, hospital-acquired infections, and decreased satisfaction. 2 8 Documentation burden impacts the work–life balance of health care providers and results from an imbalance between EHR usability and satisfaction. EHR design and use factors (e.g., information overload, slow navigation) are significantly associated with high stress and burnout among clinicians. 9 Poorer perceived EHR usability is also associated with increased burnout among physicians across specialties and practice settings. 10 Among nurses, low EHR satisfaction has accompanied reports of system-level burden (e.g., usability, interoperability). 11 Clinical and regulatory demands of entering and consuming EHR data, such as evaluation and management (E&M) services and meaningful use mandates, 3 12 13 also contribute to documentation burden.

The Quadruple Aim emerged from the Institute for Healthcare Improvement's Triple Aim framework for optimizing health system performance to address clinician burnout on health care outcomes. 14 Focusing on: (1) enhancing patient experiences, (2) improving population health, and (3) reducing costs, which outline the Triple Aim, the Quadruple Aim also includes: (4) improving health care providers' work–life balance. 14 Achieving this fourth aim would reconcile the disparity between expectations of patient-centered care and clinician capacities; however, it is contingent on several policy implications, including changes in regulatory and accreditation requirements for and practices around electronic documentation (e.g., reimbursement, quality measures). 15

During the coronavirus disease-19 (COVID-19) pandemic, health care systems nationwide promptly deployed informatics infrastructure to support clinical and operational pandemic responses (e.g., policies, procedures) at respective institutions. These actions ranged from building EHR-based tools to standardize processes (e.g., data analytics) 16 and configuring new EHR workflows, 17 to transitioning to and scaling up telehealth. 16 17 Various policies impacting clinician documentation burden were enacted, including Centers for Medicare and Medicaid Services (CMS) changes (e.g., telehealth waivers), 18 removing nonessential administrative activities, 19 and state-based relaxation of documentation requirements (e.g., recordkeeping for patient treatment and evaluation, and billing). 20 This natural experiment provides an opportunity to broadly study the impact of billing and regulatory policy “relaxations” on documentation burden, 19 which would otherwise not be achievable under conventional circumstances. The pandemic brought to the forefront the enduring tension between documentation and direct patient care, and resulted in the re-evaluation of existing practices and policies and the revival of prior documentation approaches and processes. 19 Therefore, investigating COVID-19 documentation reduction strategies is critical to the advancement of sustainable approaches to alleviate documentation-related stress, reduce clinician burnout, and improve patient safety and care quality.

Between January and February 2021, Columbia University, Vanderbilt University, and University of Virginia investigators hosted a National Library of Medicine–funded scientific meeting, convening stakeholders with the goal of reducing clinical electronic documentation burden. Leveraging Sinsky and Linzer's recommendations, 19 we developed a survey to inform the *25 by 5: Symposium to Reduce Documentation Burden on US Clinicians by 75% by 2025* (25 × 5) and generate knowledge on the impact of COVID-19 documentation reduction strategies on documentation burden.

## Objectives

We conducted a survey to assess the experiences and perceptions associated with COVID-19 documentation reduction strategies and their potential impact on documentation burden among clinicians and health care leaders. The overarching goal was to facilitate the prioritization and implementation of effective documentation reduction strategies beyond the pandemic.

## Methods

### Study Design and Data Collection

We conducted an anonymous web-based survey using Qualtrics 21 (Qualtrics, Provo, Utah) over a 6-week period from mid-November 2020 to January 2021. We recruited clinicians and health care leaders nationally to complete a self-administered survey through two channels: (1) snowball sampling via email invitations sent to and forwarded by clinicians, health care leaders, and colleagues, including professional listservs (i.e., American Medical Informatics Association [AMIA], American College of Medical Informatics, New England Nursing Informatics Consortium, Alliance for Nursing Informatics), and (2) social media (i.e., Twitter, LinkedIn, Facebook). Invitations to participate included a direct survey hyperlink. We promoted the survey through a panel presentation on documentation burden and networking sessions at the 2020 AMIA Annual Symposium. These data collection techniques broadened outreach and generalizability of data as best as possible, but did not permit calculation of a survey response rate.

### Survey

#### Survey Development

We developed a survey leveraging existing post-COVID-19 policy and practice recommendations proposed by Sinsky and Linzer. 19 The 19-item survey captured information on COVID-19 documentation reduction strategies experienced, which strategies participants preferred to remain permanent, perceptions of the strategies' potential to reduce documentation burden, and two free-text questions on additional documentation reduction strategies not described elsewhere in the survey (see Supplementary Table S1 [available in the online version only]). Our 25 × 5 Steering Committee, comprised of clinicians, informatics experts, and health care leaders, worked jointly in survey development.

##### Documentation Reduction Strategies

Eight survey items focused on *core* COVID-19 documentation reduction strategies implemented (e.g., “verbal orders permitted in hospital setting,” “telehealth expansion”). Eleven items focused on *additional* documentation reduction strategies that may have been instituted at organizations (e.g., “reduced frequency of order re-signatures,” “login optimization”). For each *core* item, participants were asked to indicate whether: (1) they “experienced the strategy,” and (2) “prefer [the strategy] to remain permanent” using checkboxes. Lastly, participants were asked to: (3) “rate the projected impact of each strategy on reducing documentation burden” based on a sliding scale from 0 (low impact) to 100 (high impact) in 10-point increments. Identical measures (outlined in 1–3) were collected for each *additional* item with the exception of “prefer [the strategy] to remain permanent,” which was replaced with asking the participant if they would “support implementing the strategy.” We added two additional free-text questions to collect information on any additional clinical documentation reduction strategies that participants experienced—related and/or unrelated to COVID-19—which were not originally captured in Sinsky and Linzer's recommendations. 19

##### Demographics

We tailored and incorporated three professional demographic questions based on fields collected by the AMIA 22 and the American Board of Medical Specialties to suit our needs: (1) profession, (2) specialty, and (3) work setting. Participants had the option of selecting up to three choices for each of the three questions using checkboxes. Location of survey completion was determined through data provided in Qualtrics.

#### Content Validity

We elicited feedback on the survey from clinical and informatics experts to determine face and content validity according to Polit and Beck's recommendations. 23 Steering Committee members identified 16 experts who were contacted directly through email, of whom half responded. Experts were asked to rate the relevance of each strategy for its ability to “assess perceptions of documentation burden reduction strategies” using a scale from 1 (not relevant) to 4 (highly relevant) and to provide overall feedback on face validity using an anonymous web-based Qualtrics 21 survey. Using the results, we calculated a content validity index on the scale (S-CVI) and item (I-CVI) level. 23 We determined that the S-CVI was 0.78, and the I-CVI ranged from 0.33 to 1. We refined our survey for clarity and incorporated additional items based on their written feedback and comments. For example, we included two additional questions on COVID-19-related practice changes that were not described in Sinsky and Linzer 19 ; these questions better captured ambulatory-specific practice changes. We also collapsed three questions on telehealth in a single item and used conditional logic that displayed detailed telehealth questions if a participant indicated they had experienced or would endorse telehealth changes. We made this decision given telehealth questions were rated with the lowest relevance by experts (0.33).

### Data Analysis

We conducted descriptive analyses on all completed surveys defined as ≥80% complete. We categorized “profession” into three mutually exclusive role categories: prescribing provider (i.e., physician, advanced practice nurse, physician assistant), registered nurse, and other. Independently, two authors (A. J. M. and J. W.) performed deductive thematic analysis on *additional* COVID-19 clinical documentation reduction experiences and any additional changes to documentation at any time that participants described in free-text based on the six domains established in the American Nursing Informatics Association (ANIA) conceptual framework on addressing burden: *reimbursement, regulatory, quality, usability, interoperability/standards, and self-imposed* (see Supplementary Table S2 [available in the online version only]). 24 Two authors (A. J. M. and J. W.) organized and reorganized discordant results until they reached a consensus on the domain(s); the domains identified were reviewed by all co-authors.

## Results

**Fig. 1:**
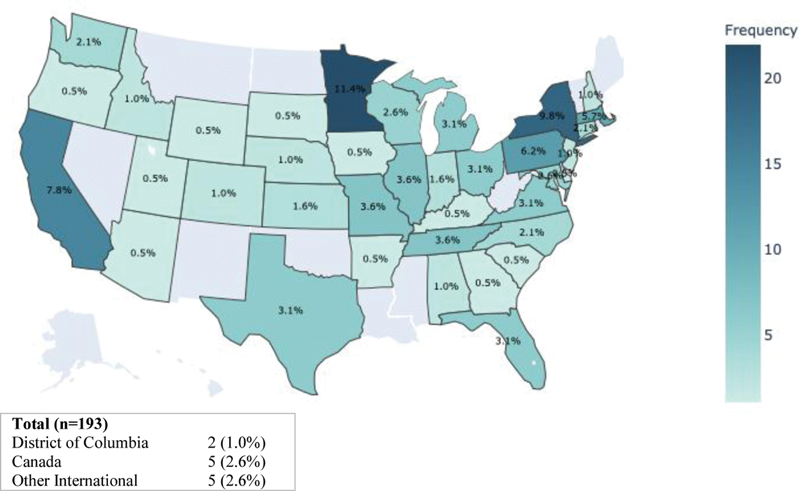
Location of survey completion by state ( *n* = 193).

**Fig. 2:**
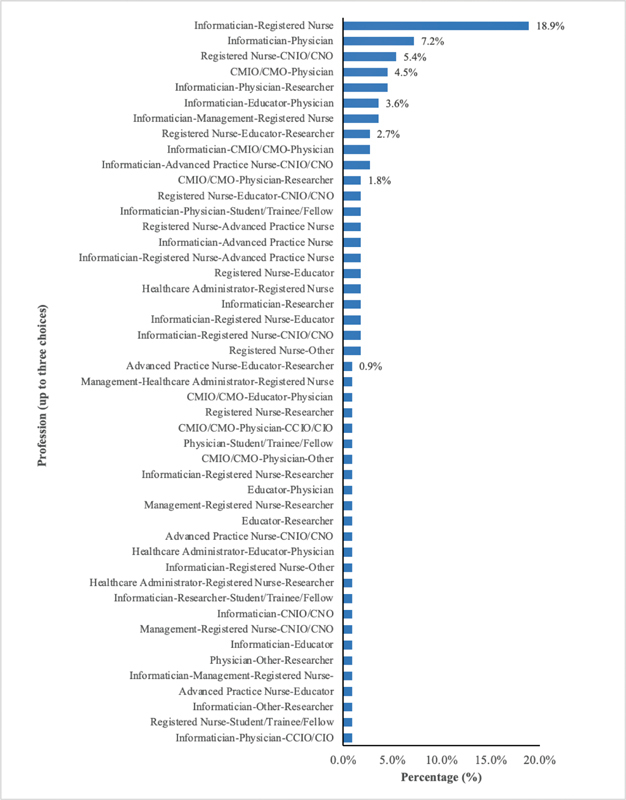
Distribution of co-occurring roles among respondents who completed the survey ( *n* = 193).

Of the 351 surveys initiated, 193 (55%) were complete. Among these surveys, most participants reported one profession (42.5%), while over a quarter reported three professions. The largest proportions of participants were informaticians (40.4%), registered nurses (36.3%), and/or physicians (34.7%) ( Table 1 ); nearly half were prescribing providers. Of the participants who reported multiple professions, informatician–registered nurse (18.9%) and informatician–physician (7.2%) were selected most frequently ( Fig. 1 ). Approximately 48% of participants worked for a health system, followed by academia (32.6%) and hospital (32.4%). The top three specialties selected were internal medicine (26.9%), family medicine (6.7%), and pediatrics (6.2%). Fig. 2 displays the geographic distribution of the survey responses for which we have the information. We received responses from participants in 37 states, including the District of Columbia, and 10 international responses. Most participants were from Minnesota (11.4%), New York (9.8%), California (7.8%), and Pennsylvania (6.2%). The following section describes results tallied among completed surveys ( *n* = 193).

**Table 1:** Professional demographics among all respondents stratified by survey completion status

Demographic variable	Complete *N* (%)	Incomplete *N* (%)	Totals *N* (%)
Total a	193 (73.1)	71 (26.9)	264 (100)
Profession b			
Informatician	78 (40.4)	37 (52.1)	115 (43.6)
Physician	67 (34.7)	16 (22.5)	83 (31.4)
Registered nurse	70 (36.3)	31 (43.7)	101 (38.3)
Chief Nursing Informatics Officer/Chief Nursing Officer (CNIO/CNO)	24 (12.4)	5 (7)	29 (11)
Researcher	22 (11.4)	6 (8.5)	28 (10.6)
Chief Medical Information Officer/Chief Medical Officer (CMIO/CMO)	19 (9.8)	5 (7)	24 (9.1)
Advanced practice nurse	20 (10.4)	5 (7.9)	25 (9.5)
Educator	20 (10.4)	9 (12.7)	29 (11)
Management	9 (4.7)	4 (5.6)	13 (4.9)
Health care administrator	6 (3.1)	2 (2.8)	8 (3)
Student/trainee/fellow	5 (2.6)	4 (5.6)	9 (3.4)
Chief Clinical Informatics Officer/Chief Information Officer (CCIO/CIO)	2 (1)	0 (0)	2 (0.8)
Physician assistant	1 (0.5)	1 (1.4)	2 (0.8)
Behavioral scientist	0 (0)	1 (1.4)	1 (0.4)
Pharmacist	0 (0)	1 (1.4)	1 (0.4)
Radiologist	0 (0)	1 (1.4)	1 (0.4)
Other	11 (5.7)	2 (2.8)	13 (4.9)
Not specified	0 (0)	0 (0)	0 (0)
Setting b			
Academia	63 (32.6)	30 (42.3)	93 (35.2)
Community-based organization	10 (5.2)	2 (2.8)	12 (4.5)
Emergency department	6 (3.1)	0 (0)	6 (2.3)
Government	9 (4.7)	2 (2.8)	11 (4.3)
Health IT vendor	14 (7.3)	7 (9.9)	21 (8)
Health plan	1 (0.5)	1 (1.4)	2 (0.8)
Health system	92 (47.7)	24 (33.8)	116 (43.9)
Hospital	66 (32.4)	23 (32.4)	89 (33.7)
Industry	8 (4.1)	8 (11.3)	16 (6.1)
Military	4 (2.1)	1 (1.4)	5 (1.9)
Nonprofit organization	18 (9.3)	7 (9.9)	25 (9.5)
Primary care	25 (13)	6 (8.5)	31 (11.7)
Private practice	5 (2.6)	0 (0)	5 (1.9)
Urgent care/walk-in clinic	2 (1)	0 (0)	2 (0.8)
Other	11 (5.7)	5 (7)	16 (6.1)
Not specified	1 (0.5)	1 (1.4)	2 (0.8)
Specialty b			
Internal medicine	52 (26.9)	13 (18.3)	65 (24.6)
Pediatrics	12 (6.2)	9 (12.7)	21 (8)
Obstetrics and gynecology	4 (2.1)	5 (7)	9 (3.4)
Emergency medicine	10 (5.2)	2 (2.8)	12 (4.5)
Psychiatry	7 (3.6)	1 (1.4)	8 (3)
Surgery	3 (1.6)	2 (2.8)	5 (1.9)
Physical medicine and rehabilitation	1 (0.5)	1 (1.4)	2 (0.8)
Radiology	0 (0)	1 (1.4)	1 (0.4)
Plastic surgery	0 (0)	1 (1.4)	1 (0.4)
Radiation oncology	0 (0)	1 (1.4)	1 (0.4)
Family medicine	13 (6.7)	0 (0)	13 (4.9)
Anesthesiology	3 (1.6)	0 (0)	3 (1.1)
Neurology	2 (1)	0 (0)	2 (0.8)
Orthopaedic surgery	2 (1)	0 (0)	2 (0.8)
Preventive medicine	3 (1.6)	0 (0)	3 (1.1)
Ophthalmology	1 (0.5)	0 (0)	1 (0.5)
Other	46 (23.8)	14 (19.7)	60 (22.7)
Not applicable	26 (13.5)	18 (25.4)	42 (16.7)
Not specified	23 (11.9)	8 (11.3)	31 (11.7)
Role categories c			
Prescribing provider	88 (45.6)	21 (29.6)	109 (41.3)
Registered nurse	66 (34.2)	30 (42.3)	96 (36.4)
Other	39 (20.2)	20 (28.2)	59 (22.3)

a Row percentages.

b Not mutually exclusive categories (participants selected up to three choices).

c Prescribing providers consist of physicians, advance practice nurses, and physician assistants.

### Quantitative Analysis

#### Core COVID-19 Documentation Reduction Strategies

##### Experience of Strategies and Preference for Strategies

**Fig. 3:**
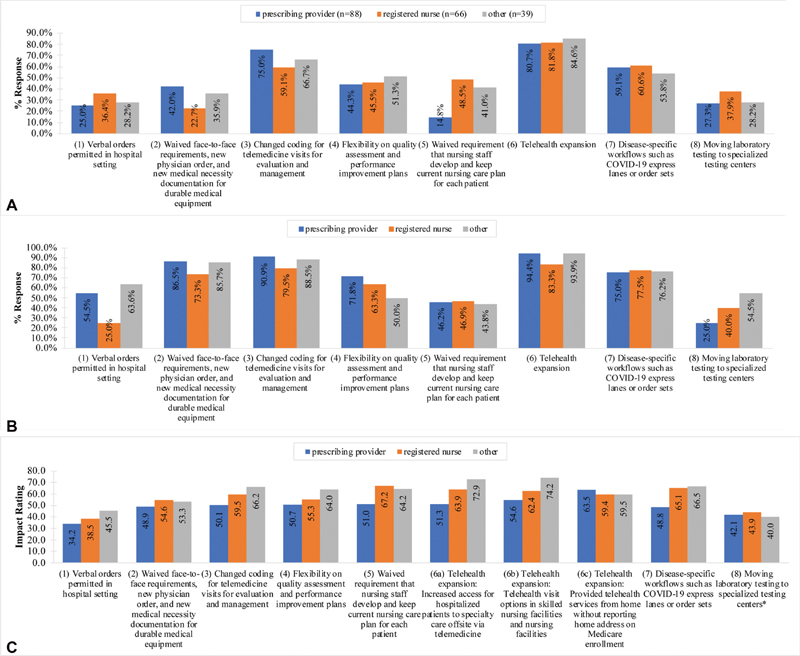
( **A** ) COVID-19 documentation reduction strategies experienced among completed surveys stratified by role category ( *n* = 193): proportion of respondents that experienced each COVID-19 documentation reduction strategy stratified by role category. ( **B** ) COVID-19 documentation reduction strategies experienced among completed surveys stratified by role category ( *n* = 193): among respondents who experienced each COVID-19 documentation reduction strategy, proportion of respondents that preferred COVID-19 documentation reduction strategy to remain permanent stratified by role category. ( **C** ) COVID-19 documentation reduction strategies experienced among completed surveys stratified by role category ( *n* = 193): average (rated) projected impact for COVID-19 documentation reduction strategy stratified by role category. *Response rates among at least one role category is <50%.

Of the eight COVID-19 documentation reduction strategies identified in the survey ( Table 2 ), most respondents experienced “telehealth expansion” (81.9%), “changed coding for telehealth visits for evaluation and management” (67.9%), and “disease-specific workflows such as COVID-19 express lanes or order sets” (58.5%); participants who experienced these strategies equally preferred that they remain postpandemic: 90.5, 87, and 76.1%, respectively ( Table 2 ). Fewer participants reported experiencing “verbal orders permitted in hospital setting” (29.5%), “moving laboratory testing to specialized testing centers” (31.1%), and “waived requirement that nursing staff develop and keep current nursing care plan for each patient” (31.6%). Similarly, these participants less preferred these strategies remain: 47.4, 36.7, and 45.9%, respectively. Few notable differences existed in the experience of and preference for these strategies between role categories except role-specific strategies (e.g., *verbal orders* , *durable medical equipment requirements* , *telemedicine coding* , *nursing care plans* ) ( Fig. 3 ). With the exception of “verbal orders permitted in hospital setting” (37.7 ± 30) and “moving laboratory testing to specialized testing centers” (42.3 ± 30.6), all COVID-19 reduction strategies (including telehealth-related subquestions) were rated 50 or more out of 100 on burden-reducing impact on average; telehealth achieved the highest average impact ratings (range: 60.1–61.5) relative to all other *core* strategies. Prescribing providers consistently rated strategies lower impact on average relative to other roles excluding *providing telehealth services from home* ( Fig. 3 ).

**Table 2:** Summary of results for documentation reduction strategies experienced among completed surveys

	Experienced strategy *N* (%)	Prefer to remain permanent *N*^a^ (%)	Projected impact of strategy *N* (%)	Projected impact of strategy		
				Mean	Median	SD
COVID-19 documentation reduction strategies ( *N*_total_ = 193)						
1. Verbal orders permitted in hospital setting	57 (29.5)	27 (47.4)	118 (61.1)	37.7	30	30
2. Waived face-to-face requirements, new physician order, and new medical necessity documentation for durable medical equipment	66 (34.2)	55 (83.3)	120 (62.2)	51.4	50	30.1
3. Changed coding for telemedicine visits for evaluation and management	131 (67.9)	114 (87)	140 (72.5)	55.8	60	33.2
4. Flexibility on quality assessment and performance improvement plans	89 (46.1)	57 (64)	126 (65.3)	54.9	60	29.6
5. Waived requirement that nursing staff develop and keep current nursing care plan for each patient	61 (31.6)	28 (45.9)	120 (62.2)	60.1	60	28.4
6. Telehealth expansion	158 (81.9)	143 (90.5)	–	–		
a. Telehealth expansion: increased access for hospitalized patients to specialty care offsite via telemedicine	–	–	134 (69.4)	60.1	60	34.4
b. Telehealth expansion: telehealth visit options in skilled nursing facilities and nursing facilities	–	–	113 (58.5)	61.4	70	32.4
c. Telehealth expansion: provided telehealth services from home without reporting home address on Medicare enrollment	–	–	112 (58)	61.5	60	30.7
7. Disease-specific workflows such as COVID-19 express lanes or order sets	113 (58.5)	86 (76.1)	141 (73.1)	57.9	60	30.3
8. Moving laboratory testing to specialized testing centers	60 (31.1)	22 (36.7)	77 (39.9)	42.3	40	30.6
	**Experienced strategy** ***N*** **(%)**	**Support** **strategy** ***N*** **(%)**	**Projected impact of strategy** ***N*** **(%)**	**Projected impact of strategy**		
				**Mean**	**Median**	**SD**
Additional documentation reduction strategies ( *N*_total_ = 193)						
1. Elimination of order requirement for low-risk activities/interventions (e.g., fingerstick glucose)	29 (15)	85 (44)	127 (65.8)	49.4	50	31.5
2. Reduced frequency of order resignatures	23 (11.9)	67 (34.7)	111 (57.5)	46.6	40	30.8
3. Documenting only pertinent positives to reduce note bloat	78 (40.4)	114 (59.1)	140 (72.5)	66.1	70	28.3
4. Increased use of documentation assistance (e.g., scribes or dictation)	60 (31.1)	81 (42)	122 (63.2)	60.6	60	28.1
5. Medication reconciliation can be performed by support staff	63 (32.6)	89 (46.1)	121 (62.7)	56.1	50	31.1
6. Changes to compliance rules and performance metrics to eliminate those without evidence of net benefit	36 (18.7)	96 (49.7)	127 (65.8)	65.7	70	26.3
7. Login optimization (e.g., badge log-ins, longer timeout interval)	68 (35.2)	113 (58.5)	136 (70.5)	56.5	60	33.3
8. Eliminate alerts without evidence of net benefit	74 (38.3)	117 (60.6)	136 (70.5)	59.7	70	31.8
9. Monitor and improve EHR use measures (e.g., pajama time)	76 (39.4)	108 (56)	124 (64.2)	60.2	65	29.3
10. EHR optimization sprints (rapid observation and improvement to EHR to meet workflow needs)	84 (43.5)	123 (63.7)	144 (74.6)	64.3	70	26.9
11. Device integration/efficient data capture (e.g., ventilators, home glucose monitoring, Bluetooth scale for heart failure exacerbations)	61 (31.6)	112 (58)	133 (68.9)	62.4	70	30.5

Abbreviations: EHR, electronic health record; SD, standard deviation.

a Denominator represents those who experienced the strategy.

#### Additional Documentation Reduction Strategies

##### Experience of Strategies and Support for Strategies

**Fig. 4:**
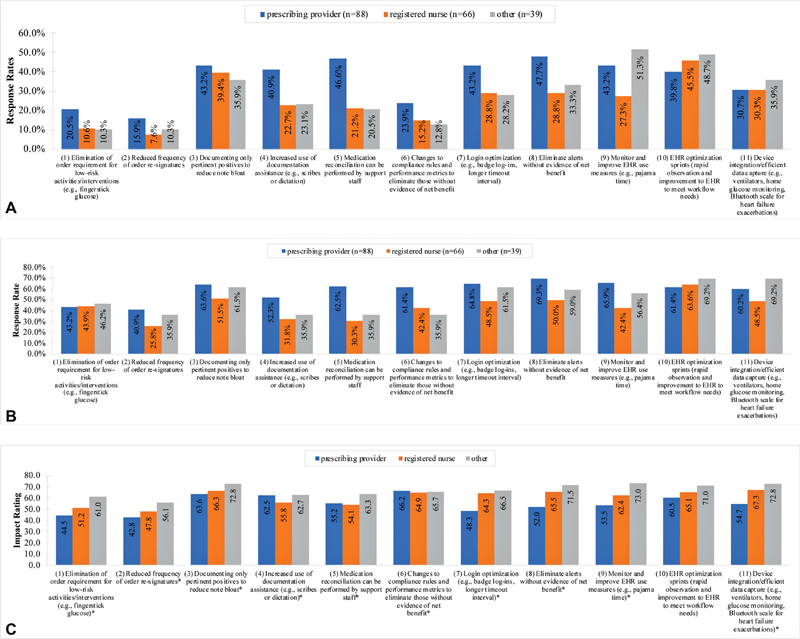
( **A** ) Additional documentation reduction strategies experienced among completed surveys stratified by role category: proportion of respondents that experienced each COVID-19 documentation reduction strategy stratified by role category. ( **B** ) Additional documentation reduction strategies experienced among completed surveys stratified by role category: among respondents who experienced each COVID-19 documentation reduction strategy, proportion of respondents that preferred COVID-19 documentation reduction strategy to remain permanent stratified by role category. ( **C** ) Additional documentation reduction strategies experienced among completed surveys stratified by role category: average (rated) projected impact for COVID-19 documentation reduction strategy stratified by role category. *Response rates among at least one role category is <50%.

Fewer than half the participants experienced each *additional* documentation reduction strategy ( Table 2 ). Participants reported experiencing “EHR optimization sprints” (43.5%), “documenting only pertinent positives to reduce note bloat” (40.4%), and “monitor and improve EHR use measures” (39.4%) at the highest proportions. Strategies including “reduced frequency of order re-signatures” (11.9%), “elimination of order requirement for low-risk activities/interventions” (15%), and “changes to compliance rules and performance metrics to eliminate those without evidence of net benefit” (18.7%) were least experienced. While participants' experience of *additional* documentation reduction strategies was low, participants supported the strategies at higher proportions. Among *additional* strategies, participants supported implementing “EHR optimization sprints” (63.7%), “eliminate alerts without evidence of net benefit” (60.6%), and “documenting only pertinent positives to reduce note bloat” (59.1%) at the highest proportions. These strategies were also rated moderately high for mean impact on burden reduction (range: 66.1–59.7). Other strategies including “changes to compliance rules and performance metrics to eliminate those without evidence of net benefit” (65.7 ± 26.3) and “device integration/efficient data capture” (62.4 ± 30.5) were less supported by participants but rated highly for impact ( Table 1 ). The least supported strategies were “reduced frequency of order re-signatures” (34.7%), “increased use of documentation assistance” (42%), and “elimination of order requirement for low-risk activities/interventions” (44%). Nevertheless, “increased use of documentation assistance” received moderately high ratings for mean impact (60.6 ± 28.1). Prescribing providers were more likely to support *additional* strategies relative to other roles ( Fig. 4 ), but consistently rated *additional* strategies lower impact on average except for *use of documentation assistance* , *medication reconciliation by support staff* , and *changes to compliance rules and performance metrics* ( Fig. 4 ).

#### Additional Experiences Reported in Free-Text Responses

Seventy participants (36.3%) identified additional experiences with clinical documentation reduction during the pandemic through free-text responses ( Table 2 ), and one-third reported additional changes to documentation at any time that had increased (64.4%) or decreased (58.6%) documentation burden. Among those, one-quarter reported experiencing both increases *and* decreases from documentation-related reduction strategies.

### Qualitative Analysis

#### Additional Experiences Reported in Free-Text Responses

We received 59 valid free-text responses regarding additional pandemic-related experiences with clinical documentation reduction. Most responses focused on the following burden domains 24 : *self-imposed* ( *n* = 30), *usability* ( *n* = 30), *quality* ( *n* = 20), and *interoperability/standards* ( *n* = 18); fewer responses centered around *regulatory* ( *n* = 10) and *reimbursement* ( *n* = 10). Responses were not mutually exclusive and often spanned multiple burden domains. 24 Eleven invalid responses comprised of survey feedback or reports of no changes.

Themes in the *self-imposed* domain included using more patient-entered data, re-evaluating system policies (e.g., instituting verbal consent, sprint teams, daily huddles), adding EHR tools to facilitate documentation workflows, and modifying documentation behaviors (see Supplementary Table S3 [available in the online version only]). *Usability* -related burden reduction strategies were similarly harmonizing documentation workflows with the EHR, employing artificial intelligence and voice recognition technologies, designing better EHR tools (e.g., templates, visualizations, autogenerated data), integrated devices for documentation, and removing alerts. *Quality* -related strategies referenced reducing documentation related to screening, care plans, and patient/family education, and charting pertinent positives. *Interoperability/standards* -based strategies involved integrated devices for documentation, eliminating note redundancies and standardizing/modifying documentation procedures, reducing data elements, and charting pertinent positives. *Regulatory-* specific strategies described adherence to telehealth, CMS, and E&M code guidelines in addition to billing requirements. Comparably, *reimbursement* strategies focused on billing requirements, for example, what is billable (e.g., telehealth, time) and whose notes are billable (e.g., medical students).

Eighty-seven participants (45.1%) reported additional changes to documentation burden at any time (i.e., not immediately linked to COVID-19). Several similar strategies reported were perceived as increasing or decreasing burden by different participants (see Supplementary Table S4 [available in the online version only]). For example, “bloated templates” and “documentation templates to document on COVID-19 confirmed cases and their discharge disposition” were identified as contributing to burden, while “smart templates to nursing admission history forms to display COVID order[s]” were reported as reducing burden. In reference to “charting pertinent positives,” one participant stated the “approach missed a lot of important information that then had to be conveyed in [the] nursing shift report and morning physician report, so in the long run it increased overall burden and decreased ability to care for patients”; another participant wrote, “[r]educe[d] screening by nurses on admission by automating record review and only bringing forward the need to assess if information not in the record” eased burden.

## Discussion

We conducted a nationwide survey among clinicians and health care leaders to assess their experiences and perceptions associated with COVID-19 documentation reduction strategies to understand which strategies should be prioritized and remain permanent post-pandemic. While some strategies were experienced at low proportions, many strategies targeting burden were imposed by health systems. We found that a majority experienced *telehealth expansion* during the pandemic *and* preferred that it remain permanent. Compared with other strategies, telehealth expansion strategies were all rated moderately high impact and over two-thirds experienced *telehealth coding changes for E&M* . While most participants preferred that these coding changes remain, participants rated these changes as slightly less impactful than individual telehealth initiatives described in the survey. These results are consistent with existing literature as the relaxation of regulations facilitated telehealth uptake and expansion, which, in turn, solved logistical challenges of simultaneously delivering care and maintaining safety during the pandemic. 25 Given these findings and well-documented inconsistencies in telehealth roll-out across institutions 26 and states 27 during the pandemic, additional efforts should be dedicated to developing a long-term regulatory framework (i.e., guidance on infrastructure, reimbursement, licensure) informed by COVID-19 experiences, eliminating barriers to expansion, 27 28 and developing telehealth platforms that are well integrated into electronic documentation workflows. 28

Less than one-third of participants experienced *moving laboratory testing to specialized testing centers* or *permitting verbal orders in hospital setting* , which were also consistent with being least preferred to remain and rated low impact comparatively. However, these strategies may not have been relevant across all participants. Low experience of and preference for strategies were not consistently linked to low impact. For example, *waiving requirements on nursing care plans for patients* had a moderately high impact rating, particularly among registered nurses.

Overall, participants were more inclined to support *additional* documentation reduction strategies that directly involved EHR usability (e.g., eliminating alerts, login optimization, EHR optimization sprints, monitoring and improving EHR use measures) and data entry (e.g., documenting only pertinent positives, device integration/efficient data capture) compared with shifting work to auxiliary staff (e.g., documentation assistance, medication reconciliation); nevertheless, contrasts between health care roles were subtle but notable. Prescribing providers were more likely to prefer *verbal orders* , and support *documentation assistance* and *medication reconciliation performed by support staff* compared with other roles, suggesting the electronic documentation ecosystem must be holistically considered when addressing burden to forestall offloading work onto other roles. *Documenting only pertinent positives* , *changing compliance rules and performance metrics to eliminate those without evidence of net benefit* , and *EHR optimization sprints* were rated highest impact compared with other strategies. Among these strategies, implementation was fairly low (range: 40.4–18.7%). As these strategies address different and interconnecting domains of documentation burden— *reimbursement, regulatory, quality, usability, interoperability/standards, and self-imposed* 24 —this implies that multifactorial solutions will be required. Despite exhibiting low implementation among participants, several *additional* strategies were supported by participants at nearly a twofold increase (e.g., eliminating alerts), suggesting the inertia may be associated with organizational culture. The optimal approach to preliminarily prioritize reduction strategies may involve targeting strategies that are highly preferred or supported *and* rated high impact, and understanding why strategies rated highly impactful were less preferred or supported.

The results of the free-text responses demonstrate that the experience of documentation burden is highly nuanced; perceptions of strategies increasing or decreasing burden pertain to who is reporting it. Templates, adding content to the EHR, and reduced documentation requirements all were described as increasing *and* decreasing burden. While many expressed “charting by exception” reduced burden, a number stated it missed important information and led to additional work, suggesting considerable variability in the perception and experience of documentation burden exists. These findings indicate that documentation reduction approaches targeting the elimination of documentation irrelevant to the clinical encounter among frontline clinicians must ensure that concision and precision do not come at a cost to the continuity of high-quality, safe patient care.

### Limitations

The survey items were based on Sinsky and Linzer's COVID-19 documentation reduction strategies 19 and those suggested by our experts, which may not be exhaustive or representative of all pandemic-related strategies implemented. 19 To mitigate this limitation, we included free-text questions to capture any unlisted documentation reduction strategies. Also, we did not evaluate survey reliability. Some strategies were irrelevant to some participants, such as “verbal orders permitted in hospital setting,” while “prefer [to] remain permanent” may be ambiguous as it does not clarify the hypothetical situation if the strategy had not already been implemented at their institution; in fact, most questions associated with preference that a strategy *remain* captured fewer responses relative to experiencing the strategies. As with all self-reported data, responses may be subject to response bias. Our sampling strategy relied on professional listservs and social media, which may not be representative of all clinicians and health care leaders who experienced clinical documentation burden; many participants identified as informaticians and were traced to five states. Due to small sample size, stratified results may not be fully interpretable ( Fig. 3 and Fig. 4 ). While this confines the generalizability of our findings, our approach was optimal for achieving a broad understanding of *burden* under rapidly evolving circumstances of the pandemic. Finally, selection bias is possible depending on whether clinical documentation burden and/or burnout influenced a participants' likelihood of survey completion. Those who identified as informaticians and/or registered nurses had proportionally more partial surveys compared with completed surveys ( Table 1 ); however, we cannot ascertain if the survey was irrelevant to these participants or if they were interrupted midcompletion.

### Future Directions

We will solicit partnerships with key changemakers to achieve the goal of reducing *overall* documentation burden by 75% over the next 5 years, which may result in documentation increases and/or decreases depending on each individual clinical context. These efforts will include reassessing the perceived impact of COVID-19 policies and others implemented to reduce burden, while considering tradeoff between data reduction and data capture. 29 Concurrent efforts must be dedicated to investigating approaches to gather clinical information without imposing on time clinicians spend engaging in direct patient care, and discovering innovative methods to apply communication and information technology (e.g., artificial intelligence, improved data models) to alleviate documentation-related stress and burnout.

## Conclusion

Natural experiments, such as the COVID-19 pandemic, provide an opportunity to broadly investigate “crisis-related policy and practice changes.” 19 Using Sinsky and Linzer's 19 recommendations, we developed and distributed an expert-validated survey to assess pandemic-related documentation reduction strategies that clinicians and health care leaders experienced. We found that a large majority experienced telehealth expansion. Compared with other strategies, participants rated telehealth strategies as highest impact on burden reduction. Subtle but notable differences were observed across health care roles. These results will inform the best approaches to decrease documentation burden in the post-COVID era.

## Clinical Relevance Statement

Increased adoption and use of EHRs have catalyzed clinical documentation burden as an issue of a national concern. Documentation burden has intensified clinician burnout and is linked to adverse effects on patient care including increased medical errors and hospital-acquired infections. The 25 × 5 Symposium assembled experts from diverse sectors to examine proximal and distal approaches for reducing and, ultimately, eliminating clinical documentation burden. The results of this survey provide insight on documentation reduction strategies implemented during the pandemic, and which strategies clinicians and other health care leaders prefer to remain, are willing to support, and deem high impact. These results will help move the needle toward achieving the Quadruple Aim.

## Multiple Choice Questions

1. Which of the following represents the domains of burden outlined in the American Nursing Informatics Association (ANIA) 24 conceptual framework to address burden in the EHR?
  - Reimbursement, regulatory, self-imposed, usability.
  - Reimbursement, regulatory, quality, usability, interoperability/standards, self-imposed.
  - Regulatory, quality, documentation, organizational, usability, interoperability/standards.
  - Regulatory, quality, usability, interoperability/standards, self-imposed, reporting.
  **Correct Answer:** The correct answer is option b. The ANIA 24 framework comprises six domains of burden that intersect at varying degrees.
2. The Quadruple Aim 14 emerged primarily to address this most recent aim:
  - Reducing costs.
  - Enhancing patient experiences.
  - Improving the work–life balance of the health care provider.
  - Improving population health.
  **Correct Answer:** The correct answer is c. The Quadruple Aim emerged from the Institute for Healthcare Improvement's Triple Aim framework for optimizing health system performance to address the growing threat of clinician burnout on health care outcomes. 14
3. Which COVID-19 documentation reduction strategy did most respondents experience *and* prefer to remain permanent?
  - Telehealth expansion.
  - Documenting only pertinent positives.
  - Documentation assistance (e.g., scribes or dictation).
  - Disease-specific workflows.
  **Correct Answer:** The correct answer is a. Over 80% of survey respondents reported experiencing telehealth expansion during the COVID-19 pandemic. Additionally, over 90% of survey respondents preferred that telehealth expansion remain permanent.

## References

[1] HealthIT.gov Strategy on reducing burden relating to the use of health IT and EHRs [Internet]Cited June 20, 2020 at:https://www.healthit.gov/topic/usability-and-provider-burden/strategy-reducing-burden-relating-use-health-it-and-ehrs

[2] Arndt B G Beasley J W Watkinson M D Tethered to the EHR: primary care physician workload assessment using EHR event log data and time-motion observations Ann Fam Med 2017 15 05 419 426 28893811 10.1370/afm.2121 PMC5593724

[3] Sinsky C Colligan L Li L Allocation of physician time in ambulatory practice: a time and motion study in 4 specialties Ann Intern Med 2016 165 11 753 760 27595430 10.7326/M16-0961

[4] Roumeliotis N Parisien G Charette S Arpin E Brunet F Jouvet P Reorganizing care with the implementation of electronic medical records: a time-motion study in the PICU Pediatr Crit Care Med 2018 19 04 e172 e179 29329162 10.1097/PCC.0000000000001450

[5] Schenk E Schleyer R Jones C R Fincham S Daratha K B Monsen K A Time motion analysis of nursing work in ICU, telemetry and medical-surgical units J Nurs Manag 2017 25 08 640 646 28853187 10.1111/jonm.12502

[6] Yen P Y Kellye M Lopetegui M Nurses' time allocation and multitasking of nursing activities: a time motion study AMIA Annu Symp Proc 2018 2018 1137 1146 30815156 PMC6371290

[7] Collins S Couture B Kang M J Quantifying and visualizing nursing flowsheet documentation burden in acute and critical care AMIA Annu Symp Proc 2018 2018 348 357 30815074 PMC6371331

[8] Overhage J M McCallie D Jr Physician time spent using the electronic health record during outpatient encounters: a descriptive study Ann Intern Med 2020 172 03 169 174 31931523 10.7326/M18-3684

[9] Kroth P J Morioka-Douglas N Veres S Association of electronic health record design and use factors with clinician stress and burnout JAMA Netw Open 2019 2 08 e199609 31418810 10.1001/jamanetworkopen.2019.9609 PMC6704736

[10] Melnick E R Dyrbye L N Sinsky C A The association between perceived electronic health record usability and professional burnout among US physicians Mayo Clin Proc 2020 95 03 476 487 31735343 10.1016/j.mayocp.2019.09.024

[11] Topaz M Ronquillo C Peltonen L M Nurse informaticians report low satisfaction and multi-level concerns with electronic health records: results from an international survey AMIA Annu Symp Proc 2017 2016 2016 2025 28269961 PMC5333337

[12] Gluckman T J Vavricek J J Streamlining evaluation and management payment to reduce clinician burden Circ Cardiovasc Qual Outcomes 2019 12 04 e005426 31001996 10.1161/CIRCOUTCOMES.118.005426

[13] Medical Informatics Committee of the American College of Physicians Kuhn T Basch P Barr M Yackel T Clinical documentation in the 21st century: executive summary of a policy position paper from the American College of Physicians Ann Intern Med 2015 162 04 301 303 25581028 10.7326/M14-2128

[14] Bodenheimer T Sinsky C From Triple to Quadruple Aim: care of the patient requires care of the provider Ann Fam Med 2014 12 06 573 576 25384822 10.1370/afm.1713 PMC4226781

[15] Bachynsky N Implications for policy: the Triple Aim, Quadruple Aim, and interprofessional collaboration Nurs Forum 2020 55 01 54 64 31432533 10.1111/nuf.12382

[16] Reeves J J Hollandsworth H M Torriani F J Rapid response to COVID-19: health informatics support for outbreak management in an academic health system J Am Med Inform Assoc 2020 27 06 853 859 32208481 10.1093/jamia/ocaa037 PMC7184393

[17] Grange E S Neil E J Stoffel M Responding to COVID-19: the UW Medicine Information Technology Services experience Appl Clin Inform 2020 11 02 265 275 32268390 10.1055/s-0040-1709715 PMC7141898

[18] HHS.gov Telehealth: delivering care safely during COVID-19 [Internet]Available at: https://www.hhs.gov/coronavirus/telehealth/index.html. Cited April 24, 2021

[19] Sinsky C Linzer M Practice and policy reset post-COVID-19: reversion, transition, or transformation? Health Aff (Millwood) 2020 39 08 1405 1411 32744939 10.1377/hlthaff.2020.00612

[20] Cuomo G A No. 202.10: continuing temporary suspension and modification of laws relating to the disaster emergencyAvailable at: https://www.governor.ny.gov/news/no-20210-continuing-temporary-suspension-and-modification-laws-relating-disaster-emergency. Cited April 24, 2021

[21] Qualtrics Qualtrics XM // The Leading Experience Management SoftwareQualtrics.2021

[22] AMIA Informatics Professionals. Leading the Way [Internet]Available at: https://www.amia.org/. Cited April 24, 2021

[23] Polit D F Beck C T The content validity index: are you sure you know what's being reported? Critique and recommendations Res Nurs Health 2006 29 05 489 497 16977646 10.1002/nur.20147

[24] Sengstack P P Adrian B David R-B Boyd L Davis A Hook M The six domains of burden: a conceptual framework to address the burden of documentation in the electronic health recordPosition Paper of the American Nursing Informatics Association Board of Directors. Available at: https://www.ache.org/-/media/ache/about-ache/corporate-partners/the-six-domains-of-burden_cerner-documentation.pdf. Cited May 1, 2021

[25] Temesgen Z M DeSimone D C Mahmood M Libertin C R Varatharaj Palraj B R Berbari E F Health care after the COVID-19 pandemic and the influence of telemedicine Mayo Clin Proc 2020 95(9S):S66 S68 32948262 10.1016/j.mayocp.2020.06.052 PMC7383140

[26] Hincapié M A Gallego J C Gempeler A Piñeros J A Nasner D Escobar M F Implementation and usefulness of telemedicine during the COVID-19 pandemic: a scoping review J Prim Care Community Health 2020 11 2.150132720980612E15 10.1177/2150132720980612 PMC7734546 33300414

[27] Lee N T Karsten J Roberts J Removing regulatory barriers to telehealth before and after COVID-10Accessed August 22, 2021 at: https://www.brookings.edu/research/removing-regulatory-barriers-to-telehealth-before-and-after-covid-19/. Published May2020

[28] Lieneck C Garvey J Collins C Graham D Loving C Pearson R Rapid telehealth implementation during the COVID-19 global pandemic: a rapid review Healthcare (Basel) 2020 8 04 517 10.3390/healthcare8040517 PMC7712147 33260457

[29] Johnson K B Neuss M J Detmer D E Electronic health records and clinician burnout: a story of three eras J Am Med Inform Assoc 2021 28 05 967 973 33367815 10.1093/jamia/ocaa274 PMC8068425

