# Supplementary material for: Clinician and Health Care Leaders' Experiences with—and Perceptions of—COVID-19 Documentation Reduction Policies and Practices
Source: Appl Clin Inform. 2021 Nov 24;12(5):1061–73. doi: 10.1055/s-0041-1739518 (PMC8612869; doi:10.1055/s-0041-1739518)
Supplement: Supplementary file 1 — Supplementary Material [file 10-1055-s-0041-1739518-s210193ra.pdf]

Supplementary Table S1 Screen captures of the survey to assess documentation reduction strategies experienced during the COVID-19 pandemic

Survey on Current Strategies for Documentation Reduction

Targeted Participants:
- Clinician (any profession or specialty)
- Healthcare/informatics leader

Survey Topics:
1) Documentation policies and practices put in place as a result of the COVID-19 pandemic
2) Strategies thought to reduce documentation burden generally

The results from this survey will inform our upcoming symposium aimed at reducing clinical electronic documentation burden titled, **"25 by 5: Symposium to Reduce Documentation Burden on US Clinicians by 75% by 2025",** co-sponsored by the *National Library of Medicine* and the *American Medical Informatics Association (AMIA)*.

Please read the IRB approved information sheet accessed using the link below which includes information on the nature and the purpose of the study as well as any risks.

By completing this survey, you confirm you have read the information sheet and consent to your responses being collected.

Please complete this survey by January 8, 2021. Thank you in advance for your contribution.

 © 2021. The Author(s).

Supplementary Table S1 (*Continued*)

Demographics: What is your profession? (Choose up to 3)

☐ Advanced Practice Nurse	☐ Registered Nurse
☐ Behavioral Scientist	☐ Occupational Therapist
☐ CCIO/CIO	☐ Pharmacist
☐ Clinical Technician	☐ Physician
☐ CMIO/CMO	☐ Physician Assistant
☐ CNIO/CNO	☐ Physical Therapist
☐ Dentist	☐ Radiologist
☐ Educator	☐ Researcher
☐ Healthcare Administrator	☐ Respiratory Therapist
☐ Informatician	☐ Speech and Language Pathologist
☐ Licensed Social Worker	☐ Student/Trainee/Fellow
☐ Management	☐ Other
	[ ]
☐ Nutritionist	

(*Continued*)

 © 2021. The Author(s).

Supplementary Table S1 (*Continued*)

Demographics: What is your specialty (if applicable)?

☐ Allergy and Immunology	☐ Otolaryngology - Head and Neck Surgery
☐ Anesthesiology	☐ Pathology
☐ Colon and Rectal Surgery	☐ Pediatrics
☐ Dermatology	☐ Physical Medicine and Rehabilitation
☐ Emergency Medicine	☐ Plastic Surgery
☐ Family Medicine	☐ Preventive Medicine
☐ Internal Medicine (including sub-specialties e.g., cardiovascular, geriatric, nephrology)	☐ Psychiatry
☐ Medical Genetics and Genomics	☐ Radiology (Diagnostic and Therapeutic)
☐ Neurology	☐ Radiation Oncology
☐ Neurological Surgery	☐ Surgery (e.g., general, vascular)
☐ Nuclear Medicine	☐ Thoracic Surgery
☐ Obstetrics and Gynecology	☐ Urology
☐ Ophthalmology	☐ Other
	________
☐ Orthopaedic Surgery	☐ Not applicable

 © 2021. The Author(s).

Supplementary Table S1 (*Continued*)

There are a number of documentation changes because of COVID-19. We would like to learn about your experiences with COVID-19 documentation reduction strategies.

Each COVID-19 documentation reduction strategy is listed on the left. After each strategy, we ask if you **experienced the strategy** and if you would **prefer that the strategy remain permanent**.

	Experienced the strategy<br>Select ALL that apply:	Prefer remain permanent<br>Select ALL that apply:
Verbal orders permitted in hospital setting	☐	☐
Waived face-to-face requirements, new physician order, and new medical necessity documentation for durable medical equipment	☐	☐
Changed coding for telemedicine visits for evaluation and management	☐	☐
Flexibility on quality assessment and performance improvement plans	☐	☐
Waived requirement that nursing staff develop and keep current nursing care plan for each patient	☐	☐
Telehealth expansion	☐	☐
Disease-specific workflows such as COVID-19 express lanes or order sets	☐	☐
Moving lab testing to specialized centers	☐	☐

(*Continued*)

 © 2021. The Author(s).

Supplementary Table S1 (*Continued*)

Please rate the projected impact of each strategy on reducing documentation burden on a scale of 0 to 100.

0 10 20 30 40 50 60 70 80 90 100

Verbal orders permitted in hospital setting

Waived face-to-face requirements, new physician order, and new medical necessity documentation for durable medical equipment

Changed coding for telemedicine visits for evaluation and management

Flexibility on quality assessment and performance improvement plans

Waived requirement that nursing staff develop and keep current nursing care plan for each patient

Disease-specific workflows such as COVID-19 express lanes or order sets

Moving lab testing to specialized centers

 © 2021. The Author(s).

Supplementary Table S1 (*Continued*)

The following are possible additional documentation burden reduction strategies that may have been instituted at your organization.

Each additional documentation reduction strategy is listed on the left. After each strategy, we ask if you have **experienced the strategy** and if you would **support implementing the strategy**.

	Experienced the strategy<br>Select ALL that apply:	Support implementing the strategy<br>Select ALL that apply:
Elimination of order requirement for low-risk activities/interventions (e.g., fingerstick glucose)	☐	☐
Reduced frequency of order re-signatures	☐	☐
Documenting only pertinent positives to reduce note bloat (e.g., charting by exception)	☐	☐
Increased use of documentation assistance (e.g., scribes or dictation)	☐	☐
Medication reconciliation can be performed by support staff	☐	☐
Changes to compliance rules and performance metrics to eliminate those without evidence of net benefit	☐	☐
Login optimization (e.g., badge log-ins, longer timeout interval)	☐	☐
Eliminate alerts without evidence of net benefit	☐	☐
Monitor and improve EHR use measures (e.g., documenting from home aka, pajama time)	☐	☐
EHR optimization sprints (rapid observation and improvement to EHR to meet workflow needs)	☐	☐
Device integration/efficient data capture (e.g., ventilators, home glucose monitoring, bluetooth scale for heart failure exacerbations)	☐	☐

(*Continued*)

 © 2021. The Author(s).

Supplementary Table S1 (*Continued*)

Please rate the projected impact of each strategy on reducing documentation burden on a scale of 0 to 100.

0 10 20 30 40 50 60 70 80 90 100

Elimination of order requirement for low-risk activities/interventions (e.g., fingerstick glucose)

Reduced frequency of order re-signatures

Documenting only pertinent positives to reduce note bloat (e.g., charting by exception)

Increased use of documentation assistance (e.g., scribes or dictation)

Medication reconciliation can be performed by clinical support staff

Changes to compliance rules and performance metrics to eliminate those without evidence of net benefit

Login optimization (e.g., badge log-ins, longer timeout interval)

Eliminate alerts without evidence of net benefit

Monitor and improve EHR use measures (e.g., pajama time)

EHR optimization sprints (rapid observation and improvement to EHR to meet workflow needs)

Device integration/efficient data capture (e.g., ventilators, home glucose monitoring, bluetooth scale for heart failure exacerbations)

 © 2021. The Author(s).

Supplementary Table S1 (*Continued*)

Please tell us about any additional experiences you have had with clinical documentation reduction during the COVID-19 pandemic.

Have you experienced any additional changes to documentation at any time that have either increased or decreased your documentation burden?

☐ Increased documentation burden:

☐ Decreased documentation burden:

The Symposium to Reduce Documentation Burden on US Clinicians by 75% by 2025 will be weekly from Friday January 15th through February 20th. Please share your email address if you would like to receive future communication about the symposium.

Thank you for your participation. Your responses will make a difference to the information presented at the Symposium.

Note: these questions were primarily derived from Sinsky C, Linzer M. Practice And Policy Reset Post-COVID-19: Reversion, Transition, Or Transformation? *Health Affairs* (Millwood, VA). 2020. doi:10.1377/hlthaff.2020.00612

Supplementary Table S2 Definitions for the six domains described in the American Nursing Informatics Association (ANIA) conceptual framework for addressing burden[24]

Domain	Definition
Reimbursement	Documentation required for payment
Regulatory	Burden associated with accreditation
Quality	Documentation required to demonstrate that patient care delivery was of high quality
Usability	Burden imposed by poor human factors engineering and design (e.g., excessive searching for information)
Interoperability/standards	Burden resulting from duplication of tasks and data entry
Self-imposed	Burden brought on by the health care organization due to culture, misinterpretation of guidelines, etc.

 © 2021. The Author(s).

Supplementary Table S3 Themes uncovered through deductive thematic analysis of *additional COVID-19 clinical documentation reduction experiences* using the six domains of burden outlined in the ANIA conceptual framework

Reimbursement	Regulatory	Quality	Usability	Interoperability/standards	Self-imposed
• Billing compliance requirements<br>• E&M[b] code guidelines on documentation<br>• Impact of telehealth on ambulatory/outpatient billing rules<br>• CMS[c] billing requirement changes<br>• Whose notes can be used for billing (e.g., medical students)<br>• Notes required for billing level	• Impact of nurse practice acts on nurse-initiated protocols<br>• Joint Commission supported policy<br>• E&M[b] code guidelines on documentation<br>• Impact of telehealth on ambulatory/outpatient billing rules<br>• CMS[c] billing requirement changes<br>• Whose notes can be used for billing (e.g., medical students)<br>• National parameters to activate "crisis documentation" needed<br>• CMS[c] recommendations for documentation during pandemic<br>• Non-patient-specific standing orders permitted by States, federal agencies, accreditation authorities, insurers, etc.	• Screening documentation and clinical calculators for SOFA[d] and mortality risk guidance<br>• "Surge" flowsheets<br>• National parameters to activate "crisis documentation" needed<br>• Reducing documentation for admission assessment (e.g., disaster documentation), patient/family education, or care plan<br>• Charting pertinent positives	• SWOT[a]/sprint teams<br>• Artificial intelligence (AI)/ voice recognition assistant to improve data entry (e.g., order entry, navigation)<br>• Designing better EHR tools (e.g., autotexts, templates, dot phrases, autogenerated data, clinical decision support, duplicate result functionalities, visualizations, information retrieval etc.)<br>• Using Design Thinking principles for EHR<br>• Tailoring EHR tools for specialties and subspecialties<br>• Adding sections in the EHR to follow a particular workflow (e.g., documentation navigators)/streamlining documentation<br>• Integrated devices for documentation (e.g., mobile devices, barcode scanning, etc.)<br>• Navigating tools not integrated in the EHR for telehealth<br>• Backup paper documentation<br>• Eliminated alerts	• Eliminate redundancies for note types<br>• Patient entered data via patient portal or office tablets<br>• Policies, guidelines, and standard operating procedures defining what not to document<br>• Streamlining workflows and autogenerated data<br>• Charting pertinent positives<br>• Reduce data element in EHR or repetition of existing data in chart<br>• Integrating data (e.g., laboratory results) from external sources to the EHR<br>• Voice recognition assistants (e.g., Dragon, Minute Note)<br>• Remote and mobile access<br>• Integrated devices for documentation (e.g., mobile devices, barcode scanning, continuous monitoring from home, etc.)	• Surveys of staff and newsletter<br>• Creating new policies, procedures, and processes for documentation workflows<br>• Changing documentation behaviors of clinicians (i.e., "defensive documentation," "if it isn't documented, it wasn't done," charting pertinent positives, fear of litigation)<br>• Re-evaluate and eliminate requirements not otherwise required by regulatory bodies<br>• Adding sections in the EHR to follow a particular workflow (e.g., documentation navigators)/streamlining documentation (e.g., assessments and screening)<br>• Training staff on full documentation requirements in EHR during reduction period<br>• Patient pre-filled data

[a]SWOT = Strengths, Weaknesses, Opportunities, Threats (analysis framework)
[b]E&M = Evaluation and Management
[c]CMS = Centers for Medicare and Medicaid Services
[d]SOFA = Sequential Organ Failure Assessment

 © 2021. The Author(s).

Supplementary Table S4 Themes uncovered through deductive thematic analysis of *additional changes to documentation at any time* (increased or decreased documentation) using the six domains of burden outlined in the ANIA conceptual framework[24]

ANIA burden domain	Increased	Decreased
**Reimbursement**	• Telehealth documentation (e.g., reason for exceptions, start and end times, time spent, registration data)<br>• Coding requirements (e.g., COVID-19 tests, specificity of ICD-10 codes[c])<br>• OpenNotes challenges due to clinical documentation inquiry (CDI) queries<br>• Problem-based charting<br>• Re-documentation of information in note	• CMS[a] rule changes<br>• Succinct templates with coding support<br>• New coding system on time<br>• Focused on medical decision making or time in clinic<br>• Training other staff to perform billing capture
**Regulatory**	• Telehealth documentation (e.g., increased nurse documentation when physician out of state, reason for exceptions, start and end times, time spent, registration data)<br>• Regulatory expectations and needs<br>• CDC[b] recommendations<br>• Emergency Use Authorization (EUA) and reporting documentation<br>• OpenNotes<br>• Rules limiting use of medical student notes	• CMS[a] rule changes
**Quality**	• COVID-19 documentation<br>• Adding content in the EHR<br>• Care plans and results of family meetings (e.g., end-of-life planning, patient education)<br>• Quality measures (with little impact on clinical care quality)<br>• Charting pertinent positives	• Eliminating documentation for patient education, second witness on medication administration, etc.<br>• Charting pertinent positives (e.g., missed documentation)<br>• Employing disaster mode documentation (e.g., assessment)<br>• Predictive models for fall and sink risk<br>• Limited dual medication sign-off for high-risk patients
**Usability**	• OpenNotes challenges due to clinical documentation inquiry (CDI) queries<br>• Alerts, pop-ups and reminders<br>• Coding requirements (e.g., COVID-19 tests)<br>• Problem-based charting<br>• Bloated templates<br>• Adding more functionalities in EHR<br>• Manual documentation of telehealth visits	• Virtual scribes and voice recognition software<br>• Auto-generated data, auto-filled notes etc. based on data within EHR<br>• Explicitly defined templates and smart templates<br>• Turning off alerts (e.g., medication interactions)<br>• Adding screen alert to reduce need to review chart for positive [COVID-19] results<br>• Improved formatting of notes/more structured clinical documentation<br>• Automating record review<br>• Succinct templates with coding support<br>• Improved flowsheets (e.g., reduced clicks)<br>• Adding sections in the EHR to follow a particular workflow (e.g., documentation navigators)<br>• Integrated devices for data capture (e.g., barcode medication administration [BCMA])<br>• Better design for review of systems (e.g., avatars)<br>• Problem-based displays in EHR
**Interoperability/Standards**	• Data re-entry for same patient	• Virtual scribes and voice recognition software<br>• Auto-generated data and auto-filled notes from data within EHR (e.g., smart template, quick visit)<br>• Explicitly defined templates and smart templates<br>• Governance structure in network to review requests for additions to EHR

 © 2021. The Author(s).

Supplementary Table S4 (*Continued*)

ANIA burden domain	Increased	Decreased
		• Screen alert to reduce need to review chart for positive [COVID-19] results<br>• "Checklists" to document care versus relying on the EHR to complete items (e.g., order entry)<br>• Adding sections in the EHR to follow a particular workflow (e.g., documentation navigators)
**Self-imposed**	• Not knowing what to document<br>• Risk managers and quality team members<br>• Adding content in the EHR<br>• Staff shortages (e.g., adding bed board) and redeployments<br>• Bloated templates<br>• Network policies and "documentation expectations"<br>• "Proving" care relative to patient outcomes<br>• Feeling the need to document irrelevant items irrespective of acuity levels<br>• Concept that EHR will fix everything<br>• Looking for the "green dot"	• Reviewed every element and eliminated items in documentation (e.g., admission assessment) of no value<br>• Return to full paper documentation<br>• Better defined terms for charting (e.g., "within defined limits")<br>• Reduced required documentation or disaster documentation standards (e.g., assessment)<br>• Piloting nurse scribes, virtual care nurse, and virtual workflow to complete checks<br>• Scribes<br>• Training other staff to perform order entry, medication reconciliation and billing capture<br>• Understanding nature of patient care and data persistence

[a]CMS = Centers for Medicare and Medicaid Services
[b]CDC = Centers for Disease Control and Prevention
[c]ICD = International Statistical Classification of Diseases and Related Health Problems

 © 2021. The Author(s).
